# Phenotypic divergence in reproductive traits of a moth population experiencing a phenological shift

**DOI:** 10.1002/ece3.865

**Published:** 2013-11-20

**Authors:** Helena M Santos, Maria-Rosa Paiva, Susana Rocha, Carole Kerdelhué, Manuela Branco

**Affiliations:** 1 Centro de Estudos Florestais, Instituto Superior de Agronomia, Universidade de LisboaTapada da Ajuda, 1349-017, Lisboa, Portugal; 2 DCEA, Faculdade de Ciências e Tecnologia, Universidade Nova de Lisboa2829-516, Caparica, Portugal; 3 INRA, CBGP, Campus International de BaillarguetCS30016, F-34988, Montferrier-sur-Lez cedex, France

**Keywords:** Egg parasitoids, egg size, fecundity, phenotypic divergence, scale covering, *Thaumetopoea pityocampa*.

## Abstract

Allochrony that is reproductive isolation by time may further lead to divergence of reproductive adaptive traits in response to different environmental pressures over time. A unique “summer” population of the pine processionary moth *Thaumetopoea pityocampa*, reproductively isolated from the typical winter populations by allochronic differentiation, is here analyzed. This allochronically shifted population reproduces in the spring and develops in the summer, whereas “winter” populations reproduce in the late summer and have winter larval development. Both summer and winter populations coexist in the same pine stands, yet they face different climatic pressures as their active stages are present in different seasons. The occurrence of significant differences between the reproductive traits of the summer population and the typical winter populations (either sympatric or allopatric) is thus hypothesized. Female fecundity, egg size, egg covering, and egg parasitism were analyzed showing that the egg load was lower and that egg size was higher in the summer population than in all the studied winter populations. The scales that cover the egg batches of *T. pityocampa* differed significantly between populations in shape and color, resulting in a looser and darker covering in the summer population. The single specialist egg parasitoid species of this moth was almost missing in the summer population, and the overall parasitism rates were lower than in the winter population. Results suggest the occurrence of phenotypic differentiation between the summer population and the typical *T. pityocampa* winter populations for the life-history traits studied. This work provides an insight into how ecological divergence may follow the process of allochronic reproductive isolation.

## Introduction

Understanding the genetic, phenotypic, and ecological differentiation occurring during speciation processes is most relevant in evolutionary biology studies. Two main types of processes are predicted to drive speciation: mutational, when genetic mutations and genetic drift dictate chance occurrence and fixation of different alleles, or ecological by adaptation to different environments or niches (Schluter 2001, 2009). Nonetheless, even when genetic mutations occur, subsequent ecological divergence due to different environmental pressures is important to complete speciation (Coyne and Orr 1998; Turelli et al. 2001; Nosil et al. 2009; Schluter 2009; Matsubayashi et al. 2010). According to the theory of ecological speciation, factors such as food availability, shelter, intraspecific competition, climate and predators can act as determinant selection pressures in the processes through which populations diverge and eventually become new species (Ridley 2004).

Reproductive isolation is usually the first step leading to genetic population divergence (Maynard Smith 1966; Orr and Smith 1998; Coyne and Orr 2004). In general, either geographical or ecological barriers commonly lead to disruption of the gene flow, causing the resulting populations to evolve under different selection pressures, so that speciation can then occur as a by-product of ecological adaptation (Schluter et al. 2001; Rolán-Alvarez 2007). Regardless of the mechanism causing two populations to split, once they become subjected to different selection pressures, ecological differentiation will reinforce the speciation process, mainly through specialization and counter-selection of hybrids (Rundle and Nosil 2005). Allochronic speciation is a particular case of sympatric speciation caused by asynchronous reproductive periods, so that individuals of different groups mate at different times of the year, season or day and consequently may become reproductively isolated. As a result, both the reproductive and the immature stages may also develop under different environmental conditions, particularly if they occur in different seasons. This type of speciation has been mainly documented in invertebrates (Marshall and Cooley 2000; Cooley et al. 2001; Abbot and Withgott 2004; Yamamoto and Sota 2009; Ording et al. 2010; Santos et al. 2011a), yet one case of sympatric speciation in a seabird species is also known (Friesen et al. 2007). Ecological divergence may thus reinforce allochronic speciation although this process has rarely been studied at population level.

The winter pine processionary moth, *Thaumetopoea pityocampa* Den. and Schiff. (Lepidoptera, Notodontidae) is a defoliator causing economic losses to pine forests in Mediterranean and other Southern European regions. The adults emerge in the summer, over a period of 30–45 days, are short lived, nocturnal, and do not feed. Females emit a specific sex pheromone, and mating is immediately followed by egg laying. The embryonic development lasts for approximately 1 month, and the gregarious larvae develop during winter time, spinning silk tents in the tree crowns between September and March. In most regions by the end of the winter, although in some areas as early as in January, the mature larvae descend to pupate in the soil undergoing an obligate diapause until the following months of July–August. In general, *T. pityocamp*a populations present this life cycle and are hereafter called winter populations (WP), referring to the time of the larval feeding period. By contrast, a unique population hereafter called the summer population (SP) was discovered in 1997 (Pimentel et al. 2006) in Leiria, Portugal, where to date, it continues to be present at high densities, having further expanded southwards and northwards along the coastline over a range of 80 km (H. M. Santos, M. Branco and S. Rocha, pers. obs.). Noteworthy is that in this forest, the SP coexists with the typical WP in the same stands and trees. Adult emergence and reproduction of the SP occur between April and June, and the larvae develop during the summer and pupation occurs in September, thus markedly differing from the life cycle of the WPs (Pimentel et al. 2006).

Seasonal adult activity was studied for 3 years (2005, 2007, and 2008), and results demonstrated the occurrence of an interval of 24–41 days between the end of the reproductive period of the SP and the onset of that of the sympatric WP, hence causing reproductive isolation by time (Santos et al. 2007). Males of both populations are attracted to the same synthetic pheromone, and the composition of the female sex pheromone, namely (Z)-13-hexadecen-11-ynyl acetate (Guerrero et al. 1981), is similar (Branco et al. in press). Thus, olfactory communication and male sexual attraction remain potentially viable between the SP and the WP. Sequencing of the mitochondrial cytochrome oxidase 1 and of the nuclear ITS1 marker showed that the two sympatric Leiria populations have recently separated. Further, the analysis of microsatellite markers suggested that the SP was founded by individuals originating from the local WP and that the intensity of gene flow between the two populations is now severely reduced (Santos et al. 2007, 2011a).

Due to its unique phenology, each life stage of the *T. pityocampa* SP faces environmental and ecological conditions different from those experienced by the WP, in particular regarding climatic conditions and pressures from natural enemies. In a previous study, Santos et al. (2011b) reported that for the young larvae of the SP, the upper threshold of survival in relation to temperature was situated above 40°C, whereas for several populations of the WP, it remained below 36°C, thus indicating a better adaptation of the SP to high temperatures.

This work aimed at assessing the occurrence of ecological divergence between the SP and the WP, focusing on the characteristics of the eggs and of the egg batches, by targeting several morphological traits that may affect embryonic survival, as well as differences between the communities of the egg parasitoids. Several parasitoid species are known to attack the eggs of *T. pityocampa*, mainly the specialist *Baryscapus servadeii* (Dom.) (Hymenoptera, Eulophidae) and two polyphagous species *Ooencyrtus pityocampae* (Mercet) (Hymenoptera, Encyrtidae) and *Trichogramma embryophagum* (Hartig) (Hymenoptera, Trichogrammatidae) (Biliotti 1958; Tiberi 1990; Tsankov et al. 1996, 1999; Schmidt et al. 1997, 1999; Mirchev et al. 2004). As the eggs of the SP and of the WP are present in the field in different seasons, it can be expected that the activity of the specialist egg parasitoids might not be synchronized with the occurrence of the SP, thus favoring low parasitism rates in the SP. Furthermore, as generalist parasitoids use alternative hosts and are active over longer periods of the year than the specialist parasitoids (Battisti et al. 1988), the former should account for a higher percentage of the total parasitism.

The females of *T. pityocampa* lay their eggs wrapped around one or two pine needles, in a single egg batch, protected by the deposition of abdominal scales (Schmidt et al. 1999). It is plausible that egg covering should be linked to an ecological function enhancing egg hatching success. The scales cover affords protection from abiotic factors, such as cool air temperature and insolation (Milani 1990), as well as from biotic factors like parasitoids and other natural enemies (Schmidt 1990; Pérez-Contreras and Soler 2004). However, both types of factors may strongly differ between the spring and the summer seasons, and therefore, unequal investments in egg covering could be made by the females of the two populations, in response to the different seasonal pressures.

At an evolutionary scale, differences in natural selection among environments, including climate and pressure from natural enemies, can influence female fecundity and egg size (Fox and Czesak 2000). Both traits directly affect fitness although a trade-off between them is usually observed (Fox and Czesak 2000; Czesak 2003; Fischer et al. 2003; Pérez-Contreras and Soler 2004). In typical WPs, the number of larvae per colony, which is proportional both to female fecundity and to the number of egg clusters that join to form colonies, strongly affects colony survival during the winter (Pérez-Contreras et al. 2003). However, as the larvae of the SP develop under warmer temperatures than those of the WP (Pimentel et al. 2006), colony size in the former population will not necessarily affect larval survival as much as in the WPs. Further, egg size determines the amount of reserves available for the embryo to feed and grow and consequently affects the size of the neonate larvae (Fischer et al. 2003). Selection might thus favor egg batches having a lower number of larger eggs, whenever colony size does not act as a limiting factor.

In this work, the following phenotypic traits concerning the egg stage were compared for the SP and for several WPs: (1) realized female fecundity, (2) egg size, (3) egg batch covering (scale dimensions, shape and color), (4) rates of parasitism, and (5) composition of the parasitoid communities. Several WPs were studied in order to estimate the occurrence of variability among them, as well as to ascertain if the parameters studied for the Leiria WP could also be considered representative for other *T. pityocampa* populations of Portugal. We considered a latitudinal cline based on findings of Pimentel et al. (2010), who reported that, in *T. pityocampa,* fecundity increases and egg size decreases with increasing latitude. Considering that divergent evolutionary processes can be expected to increase the phenotypic distance between sympatric populations (Coyne and Orr 2004), we aimed at testing if this unique, phenologically divergent population, which is subjected to climatic and biotic pressures different from those faced by the ancestral population, would present phenotypical differentiation in the egg stage. Findings would provide support for the hypothesis of ecological divergence and possible reinforcement, following allochronic reproductive differentiation.

## Materials and Methods

### Sampling design

Egg batches from eight *T. pityocampa* winter populations (WPs, including Leiria WP) were collected between 2000 and 2011, from *Pinus pinaster* stands distributed in Portugal along a latitudinal gradient, as well as from the Leiria summer population (SP) (Table 1). The fresh egg masses were collected just before, or shortly after egg hatching. The egg batches were primarily not specifically collected for this study, thus explaining their uneven distribution across years, depending on the populations. A total of 759 egg batches from nine populations were finally analyzed. All stands sampled had a similar tree density of about 1300 pines per ha.

**Table 1:** Populations of *Thaumetopoea pityocampa* studied: location and coordinates of the pine stands, years, and number of egg batches collected. Portugal 2000–2009.

Population	Coordinates (lat, long)	Elevation (m)	Years of sampling	*N*. of egg batches
Alcácer do Sal	38°23′N; 08°31′W	50	2002	12
Apostiça	38°30′N; 09°11′W	35	2000; 2003; 2005	163
Rio Frio	38°40′N; 08°52W	20	2006	23
Azambuja	39°05′N; 08°53W	100	2006	23
Barrada	39°25′N; 08°03W	150	2007	13
Abrantes	39°33′N; 08°14′W	160–230	2000; 2003	176
Leiria SP	39°50′N; 08°55′W	30–50	2000; 2004; 2006; 2007; 2008; 2009; 2010; 2011	283
Leiria WP	39°50′N; 08°55′W	30–50	2000; 2004; 2005	36
Vila Real	41°19′N; 07°44′W	480	2003	30

Using as indicator the number of males caught in pheromone traps, set up to assess the phenology of *T. pityocampa* (see below), it was possible to estimate the density of both the Leiria SP and the WP, in 2005 and 2007, but not of the other populations.

Regarding the parameters fecundity, rates of parasitism and frequency of occurrence of each parasitoid species, it was possible to obtain reliable data for all the studied PPM populations and egg batches collected. However, due to methodological difficulties, we were unable to acquire enough data regarding egg size, scales characteristics, phenology of *T. pityocampa* and of the associated egg parasitoids for all the studied populations. The study on regional variability was thus based on three populations only: the two sympatric, Leiria SP and WP, plus another WP from Apostiça. The later was chosen due to its location, ca. 150 km south from Leiria, at the same elevation and longitude, presenting a phenology similar to the Leiria WP, while previous data showed that genetic differentiation between them is very limited (Santos et al. 2011a).

### Female fecundity and egg size

The number of eggs per egg batch was counted in the laboratory under a binocular microscope, for all egg batches and used as a proxy for realized female fecundity.

Egg size was calculated for 10 egg batches per population for the Leiria SP and WP and for Apostiça. For each egg batch, 10 eggs were measured, using one–two eggs per line along the length of the egg batch. The measurements were made using the minor axis of the elliptical eggs using a binocular microscope (100× magnification) and an ocular micrometer, following the procedure described by Zovi et al. (2008). An indicative estimate of egg volume was calculated by considering that egg shape could roughly be considered spherical and using the parameter egg size as diameter.

### Egg parasitism

Egg parasitism was quantified for the nine *T. pityocampa* populations studied by keeping egg batches in the laboratory separately, inside vials until emergence of the parasitoids ceased. All parasitoids that emerged were collected, identified, and counted. The eggs were then uncovered by removing the scales, and all hatched, unhatched, and parasitized eggs were counted.

As some parasitoids could have emerged in the field before egg batch collection, the number of parasitized eggs (and thus, parasitism rate) was determined by counting the number of eggs with emergence holes of parasitoids. These data were used for the parasitism analyses, while the number and species of parasitoids that emerged in the laboratory, either in the current year or after undergoing the winter diapause, were used to determine the species frequencies. Although the parasitoid community analyzed is a subsample of the whole parasitoid community, identical methodological procedures were adopted for all populations.

### Egg parasitoids and *Thaumetopoea pityocampa* phenology

According to Battisti (1989) and Schmidt et al. (1999), the egg parasitoids of *T. pityocampa, B. servadeii,* and *O. pityocampae* may have a first generation without diapause, with adults emerging about 2–3 weeks after oviposition, while host eggs are still available. A second generation can thus be initiated that will undergo a diapause and emerge in the following year, coinciding with either the next egg laying season of the pine processionary moth, or with the laying period of alternative hosts in the case of generalist parasitoids. The period of *T. pityocampa* male flight was monitored by pheromone trapping, characterized and used as a surrogate for egg laying (i.e., availability of egg batches in the field). The emergence of the diapausing parasitoids from the collected egg batches was monitored, to test for synchronization between both parasitoid species and the egg laying periods of the SP and of the WP. Such experiments were conducted in Leiria (using parasitoids emerged from both the SP and the WP) and Apostiça. Adult males were sampled in the field throughout the flight season, in 2005 and 2007, using funnel traps baited with dispensers containing synthetic female sex pheromone (pityolure 40 mg), as described in the study by Santos et al. (2007).

### Scales

Scale removal from the egg batches proved a difficult task, as the scales are strongly glued to the egg shell and most of them were broken in the process. As the scales applied by the females to cover the egg batches are taken from the tip of the abdomen (H. M. Santos, pers. obs.), we adopted the procedure of removing them directly from virgin females that emerged in the laboratory. These originated from mature larvae of field populations collected in Apostiça, Leiria SP, and Leiria WP that pupated and emerged in the laboratory. Twenty females were sampled from different colonies of each population, each female supplying eight to 12 scales that were mounted between two microscope cover slips and fixed with tape. The preparations were then scanned, and the images analyzed using the software WinSEEDLE™ 2008 (Regent Instruments Inc., Quebec, Canada). Four biometric variables calculated by this software were selected as follows: (1) Area of the scale (A – in mm^2^), (2) form coefficient (Form = 4πA/P^2^ where A = scale area and *P* = scale perimeter in mm). Form varies between 0 and 1, 1 being a perfect circle and 0 a filiform object, (3) maximum straight width (W – in mm), and (4) width to length ratio (W/L, L being the maximum straight length in mm).

The WinSEEDLE software works with up to 12 color classes defined by the user, divided into groups of colors. Twelve color classes, representative of the color range found in the scales sampled, were thus selected, and three groups of colors (light, medium, and dark) were then defined, each group including four color classes. This software calculated the percentage of the area occupied by each class, assigning each pixel to one of the 12 classes defined. Orphan pixels (those that do not match exactly to any of the defined classes) were automatically assigned to the closest class. Data resulting from this analysis were further aggregated into two mutually exclusive groups: dark colors and light colors. Results were expressed as percentage of the scale area represented by light colors.

### Data analysis

Data obtained from the egg batches were analyzed using SPSS statistics version 17.0 (IBM Corporation, Armonk, NY) and presented as mean ± standard error of the mean (SEM).

#### Realized female fecundity

The number of eggs per egg batch was used as an indicator of the realized female fecundity. This variable was analyzed for the nine populations by generalized linear models (GLM), using a Poisson log linear link function, considering the population factor. A maximum likelihood estimation procedure was used. Differences between populations were analyzed using a least significant differences (LSD) test (α = 0.05). As we were not able to explicitly take the variable time into account in the procedure described above, although interannual variability of the environmental conditions can affect female fecundity, we conducted the same analysis to compare only the Leiria SP with the Leiria WP, using the data obtained for the two common years of sampling, 2000 and 2004. The factors year and population were considered, as well as their interaction.

To relate the egg load with latitude and check whether the latitudinal gradient identified by Pimentel et al. (2010) would also occur at this smaller spatial scale, the geographical coordinates for each population were transformed into rectangular coordinates using the military Datum Lisboa system and correlated using Spearman's coefficient, *ρ*, with the mean values of each parameter for all populations. This analysis was conducted using all sampled WPs.

#### Egg size

A linear mixed model was used to analyze differences between populations regarding egg size. The eggs measured within the same egg batch were considered as repeated measurements, egg batches as subjects, and populations as factors.

#### Parasitism

The percentage of parasitized eggs was calculated and analyzed by GLM, after application of the transformation ln(x + 1), with normal link function, considering the predictor variable population. A maximum likelihood estimation procedure was used and an LSD test applied, to compare population means (α = 0.05). Due to the constraints explained above, the same procedure was used to compare the two sympatric populations, Leiria SP and Leiria WP, for 2000 and 2004, considering the factors year and population, as well as their interaction. Yet, parasitism rates may be affected by overall host density, which must be recalled for result interpretation.

To test for differences between the two reproductively isolated populations regarding the frequencies of the parasitoids, grouped as specialists and generalists, a Pearson's chi-squared test was used.

#### Scales shape and color

The five variables measured from the scales, namely the four biometric estimates and the color classes, were treated by a principal component analysis (PCA) to differentiate groups. A discriminant analysis was then performed using the same five variables and the three predetermined groups (Leiria SP, Leiria WP, and Apostiça). Two discriminant functions were estimated to separate the three groups. The probability of each individual being correctly assigned to its group was further determined.

## Results

### Realized female fecundity

The number of eggs per egg batch varied between 16 and 322 with significant differences among populations (Wald chi-square = 2335.5, df = 8, *P* < 0.001). Of 759 egg batches studied, eight only (including five from Leiria SP) contained less than 50 eggs. The lowest mean fecundity (139.4 ± 3.37) was observed in Leiria SP, this value being significantly lower than in all winter populations studied (*P* < 0.001). For the WPs studied, the lowest fecundity was found in Rio Frio, yet still differing significantly from the Leiria SP by 11.42 ± 2.70 (*P* < 0.001) eggs per egg batch. By contrast, among the WPs, the sympatric Leiria WP had the highest mean number of eggs per batch (195.7 ± 8.90). Vila Real, the northernmost population studied, had the second highest fecundity which was not significantly different from the Leiria WP (*P* = 0.069).

A comparison between the sympatric Leiria populations, SP and WP, using data for 2000 and 2004, detected significant differences regarding the factors population (Wald chi-square = 553.47, df = 1, *P* < 0.001) and year (Wald chi-square = 67.96, df = 1, *P* < 0.001), but not for the interaction, year*population (*P* = 0.930). Consistently, for both years, fewer eggs per egg batch (134.1 ± 1.40) were observed in the Leiria SP, than in the Leiria WP (197.7 ± 2.53).

No significant correlation was found between the mean number of eggs per batch and latitude (*ρ* = 0.45; *P* = 0.26).

### Egg size

Egg size was significantly higher in the Leiria SP (1.22 ± 0.004 mm) than in both WPs from Leiria (1.16 ± 0.004 mm) and Apostiça (1.07 ± 0.005 mm) (*F*_2,273_ = 314.25; *P* < 0.001) although the two winter populations differed significantly between them (*P* < 0.001). The ratio egg size/egg load did not differ significantly among populations (*F*_1,2_ = 1.94; *P* = 0.163).

Egg volumes of 0.95 ± 0.010, 0.82 ± 0.009, and 0.65 ± 0.009 mm^3^ were estimated, respectively, for Leiria SP, Leiria WP, and Apostiça WP, reflecting the differences in the amount of reserves available for the development of the embryos.

### Parasitism

**Figure 1:**
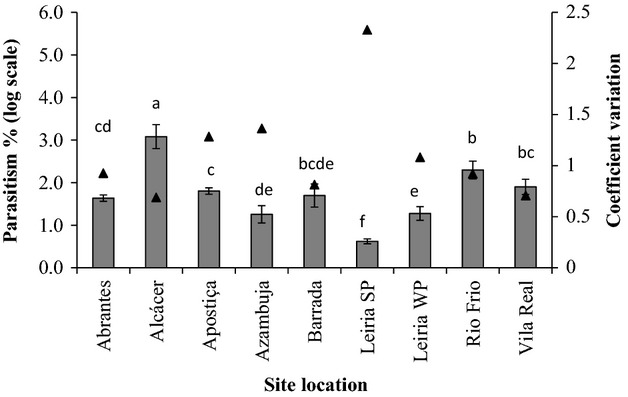
Mean parasitism rate (%) ± SE (bars) and coefficient of variance (CV) of the parasitism rates (dots) for the egg batches of eight *T. pityocampa* winter populations (WPs) and for the Leiria summer population (SP).

The percentage of parasitized eggs per egg batch differed significantly between populations (Wald chi-square z value = 182.59, df = 8, *P* < 0.001). Model deviance was close to one (0.96). The lowest mean parasitism rate was observed in the Leiria SP and the second lowest in the Leiria WP (Fig. 1). The corresponding coefficient of variation (CV) was highest in Leiria SP (2.3), whereas it approached one for all other populations (Fig. 1). A comparison of the two sympatric Leiria populations, SP and WP, for the years of common sampling (2000 and 2004), showed that none of the factors analyzed, population (*P* = 0.119), year (*P* = 0.588), and interaction year*population (*P* = 0.554) was significant.

The mean rates of parasitism (%) observed over 8 years of sampling (Table 1), varied in the Leiria SP, between 0 ± 0 (*n* = 27) in 2011 and 4.9 ± 1.3 (*n* = 38) in 2004, and in the Leiria WP, between 2.0 ± 0.5 (*n* = 4) in 2005 and 5.3 ± 1.7 (*n* = 13) in 2004. For the populations from the other studied regions, mean parasitism rates varied from 5.3 ± 1.5 (*n* = 23) in 2006, in Azambuja, to 28.9 ± 3.5 (*n* = 12) in 2002, in Alcácer do Sal.

**Figure 2:**
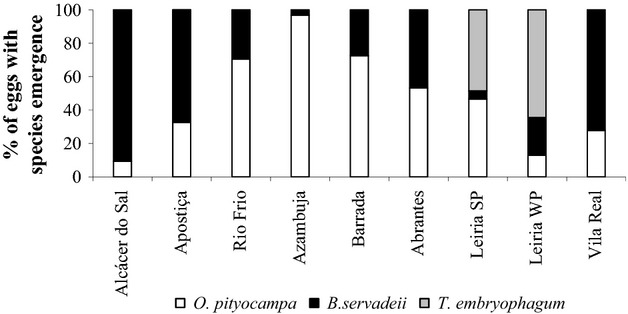
Proportion of egg parasitoid species emerged for eight *T. pityocampa* winter populations (WPs) and for the Leiria summer population (SP).

**Figure 3:**
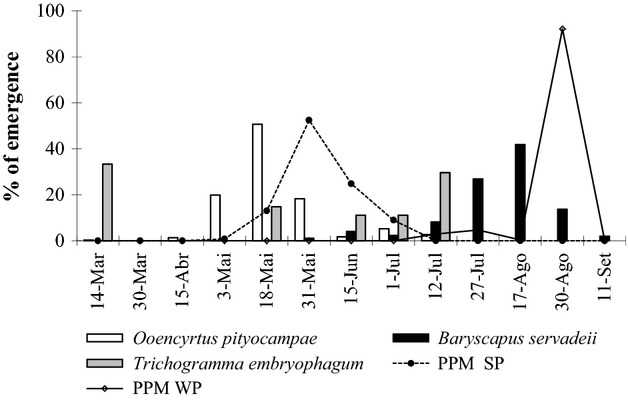
Phenology of the egg parasitoid species of *T. pityocampa*, observed for the Leiria summer population (SP) and adult flight periods of both Leiria SP and Leiria WP.

*Ooencyrtus pityocampae* and *B. servadeii* were the main parasitoid species found in all samples, except in those from Leiria although the relative abundance of the two species varied among populations. High proportions of *T. embryophagum* were observed in both Leiria populations, SP and WP, but not elsewhere (Fig. 2). *Ooencyrtus pityocampae* was abundant in the Leiria SP, representing 46.6% of the total parasitism, a value close to the observed frequency of *T. embryophagum* (48.6%). The two Leiria populations differed significantly regarding the frequency of the generalist species *O. pityocampae* and *T. embryophagum* when data were pooled, by comparison with the specialist *B. servadeii* (Chi-square = 76.87, df = 1, *P* < 0.001). *Ooencyrtus pityocampae* started emerging early in the season, having a main peak of emergence between March and June, synchronous with the period of male flight for the SP. *Baryscapus servadeii* was the second most important species parasitizing the Leiria WP (20%), with emergences well synchronized with the male flight period of this population and marginally overlapping with the flight period of the SP (Fig. 3). *Trichogramma embryophagum* emergence occurred over a wider period, as adults were observed from the end of the winter up to the beginning of the summer.

The number of males caught per trap, during the complete flight seasons of 2005 and 2007, was for the Leiria SP, respectively, 8.8 ± 3.0 (*n* = 17) and 20.0 ± 7.5 (*n* = 12) and for the Leiria WP, respectively, 0.4 ± 0.3 (*n* = 18) and 1.2 ± 0.6 (*n* = 15).

### Scales shape and color

All morphometric and color variables estimated for the scales differed significantly among populations, with Leiria SP always presenting the lowest values (Table 2). Furthermore, the phenotypic distances between Leiria WP and Leiria SP were always larger than between the two WPs of Leiria and Apostiça (Table 2). The area and color of the scales did not differ significantly between both WPs.

**Table 2:** Pairwise comparisons using LSD (ANOVA) for the egg scales variables (Mean ± SE, *n* = 10 × 20 females/population) of three *Thaumetopoea pityocampa* populations: different letters indicate significant differences at *P* < 0.05.

Variable	Area	Straight width	Width/Length ratio	Form coefficient	Color
Leiria SP	3.04 ± 0.11^a^	1.22 ± 0.04^a^	0.40 ± 0.01^a^	0.60 ± 0.01^a^	44.51 ± 2.12^a^
Leiria WP	3.69 ± 0.11^b^	1.57 ± 0.04^b^	0.52 ± 0.00^b^	0.67 ± 0.01^b^	67.10 ± 2.12^b^
Apostiça	3.84 ± 0.11^b^	1.69 ± 0.04^c^	0.58 ± 0.01^c^	0.73 ± 0.01^c^	67.04 ± 2.12^b^
*F*_2,57_	15.93	48.99	86.74	50.81	37.91
*P*	<0.001	<0.001	<0.001	<0.001	<0.001
Phenotypic distance					
Leiria WP – Leiria SP	0.65	0.35	0.12	0.07	23.39
Apostiça – Leiria WP	0.15	0.12	0.06	0.06	0.06
Apostiça – Leiria SP	0.80	0.47	0.18	0.13	22.53

**Figure 4:**
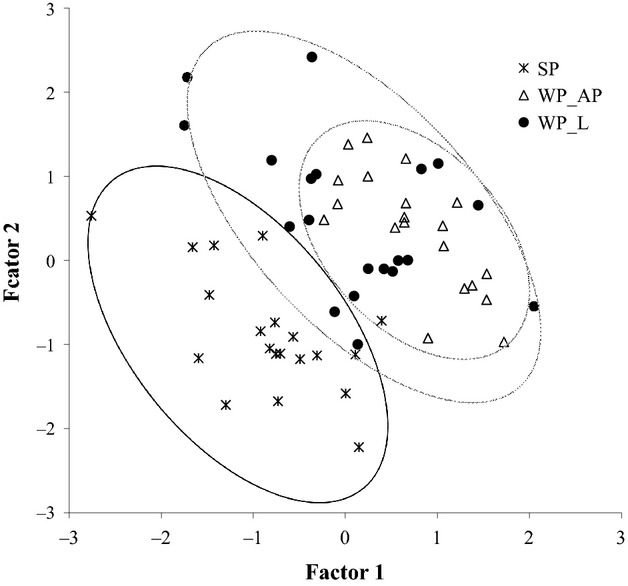
Principal component analysis (PCA) using four phenotypic variables (area; straight width, width to length ratio, form coefficient, see text for details) and color of the egg scales of *T. pityocampa*, based on mean values (*n* = 10 × 20 females/population). The first and second PCA components explain 92% of the variance. Ellipses enclose populations for better visualization of the separation.

Results of the multivariate PCA demonstrated that the Leiria SP is separated from the two winter populations, Leiria WP and Apostiça, while the two later overlap (Fig. 4). Furthermore, a higher variability was observed in both Leiria SP and Leiria WP than in Apostiça (Fig. 4). Using discriminant functions, it was possible to separate the three populations based on the scales phenotypic measurements (Chi-square = 97.172, df = 10, *P* < 0.001 and 9.018, df = 4, *P* = 0.061, for the first and second discriminant functions, respectively). Concerning the Leiria SP, 95% of the observations were placed in the same group (one observation of 20 was assigned to the Leiria WP). For the Leiria WP, 85% of the observations were correctly assigned (one was wrongly assigned to Leiria SP and two to Apostiça). Regarding Apostiça, 80% of the observations were correctly classified (four observations were assigned to the Leiria WP).

**Figure 5:**
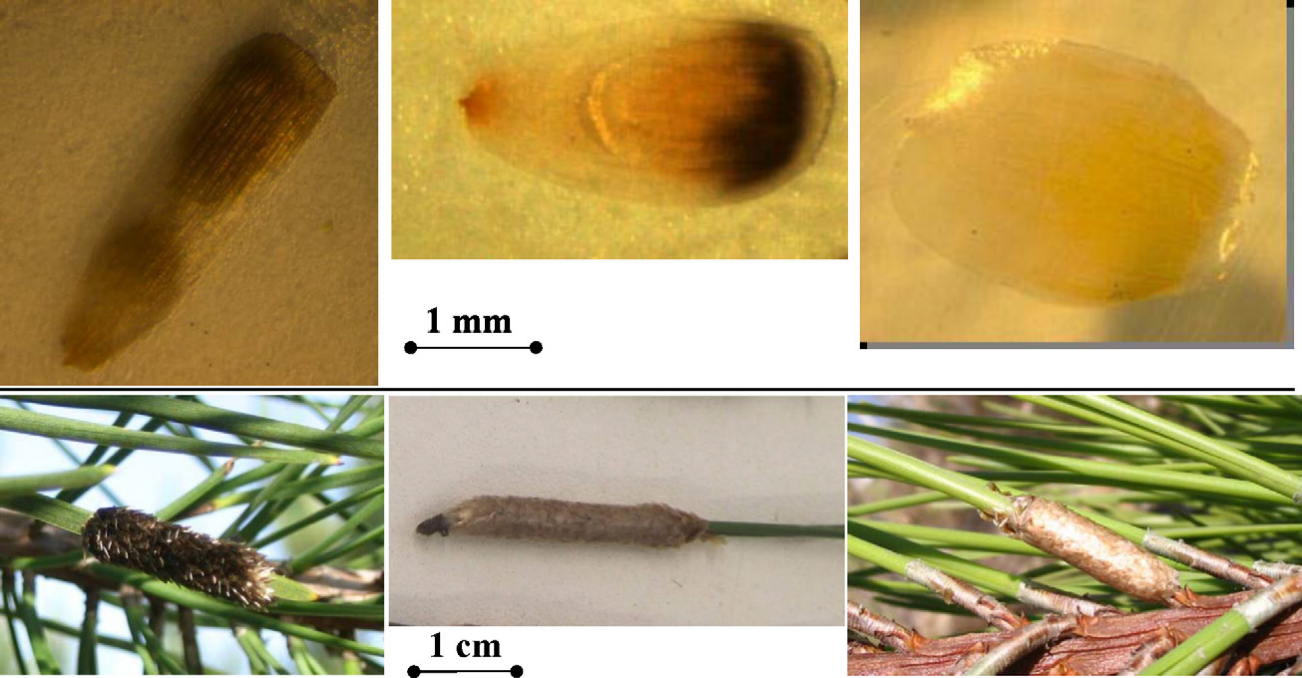
Detailed images of the abdominal scales of *T. pityocampa* (above) and of the egg batches (below). Leiria summer population (left), Leiria winter population (center) and Apostiça winter population (right).

The scales of the Leiria SP were, on average, darker, thinner, and smaller than those of both WPs, having an accentuated triangular shape, a form coefficient of ca. 0.60 (±0.01) and a length to width ratio of 2.5 (Table 2, Fig. 5).

## Discussion

The results presented allow us to conclude that the reproductive traits of the *T. pityocampa* allochronic population (SP) discovered in Leiria are phenotypically different from those of the typical WPs, originating either from Leiria or from seven other studied regions of Portugal.

Santos et al. (2011a) obtained data supporting the hypothesis that the SP has relatively recently evolved from the sympatric ancestral WP. Theoretically, the differences observed between the SP and the typical populations could have resulted either from local genetic adaptation, phenotypic plasticity, or drift following a founder effect in Leiria forest. Independently of the mechanism causing these phenotypic differentiations, the life stages of the SP develop in distinct seasons as compared with the WPs, thus becoming subjected to different selective pressures.

### Egg size and fecundity

In our study, latitude did not influence the female fecundity of *T. pityocampa* WPs, suggesting that the gradient observed by Pimentel et al. (2010) does not occur at this spatial scale, when sampling was always conducted on the same host pine. Among the nine populations studied, Leiria SP had the lowest number of eggs per batch. Although variability across sampling years and possibly also among population densities may act as potential confounding factors for data interpretation, consistent results were still achieved when comparing the two sympatric populations. As the number of eggs per batch was significantly lower in the Leiria SP than in the WP, for both years of common sampling, the observed trends may be considered as fairly consistent.

The lower fecundity observed in Leiria SP was compensated by larger eggs, by comparison with all WPs, thus illustrating a trade-off between egg size and fecundity which is well established in the literature (Fox and Czesak 2000; Fischer and Fiedler 2001; Gillooly et al. 2002; Fischer et al. 2003; Pérez-Contreras and Soler 2004; Pimentel et al. 2010). Survival of the early larval instars is usually favored by larger eggs, and thus higher neonate larval size (Fox et al. 1997; Fox and Czesak 2000). Due to the gregarious larval behavior of *T. pityocampa*, in WPs, the number of eggs in an egg batch can have a direct influence on colony size and family survival during the winter (Pérez-Contreras et al. 2003). Nevertheless, larvae from different egg batches are known to merge into a single colony in the same tree when population densities are high (Branco et al. 2008). As the larvae of the SP are not exposed to low winter temperatures as development takes place mostly during the summer, the number of larvae per colony is probably not as critical as it is for the WPs. In accordance, a lower investment in silk production is made by the larvae, so that the tents built by the SP are unstitched structures (Authors, pers. obs.). On the other hand, it may be hypothesized that a lower investment in silk production could render the colony more vulnerable to predators. Embryonic development in the SP occurs at lower temperatures (mean monthly temperature in May = 15.6°C, data 2001–2011, from http://www.snirh.pt) than in the WP (mean monthly temperature in August = 19.7°C). Larger eggs may thus be favored because they probably improve embryonic survival in colder environments, as documented in another Lepidoptera species (Fischer et al. 2003). Moreover, larger eggs produce larger neonate larvae that may survive better on the main host *P. pinaster*, as this pine species has tough needles that cannot be consumed by the smallest caterpillars (Zovi et al. 2008). Furthermore, the larvae of the SP mostly consume tougher needles from the previous year, in preference to the softer ones of the same year (Authors, pers. obs.) although hatching coincides with the development of the new shoots. In conclusion, it may be postulated that, for *T. pityocampa* SP, the combined effect of different mechanisms (embryonic and neonate survival, absence of counter-selection due to cold winter temperature) could favor egg masses with a smaller number of larger eggs, by comparison with the WPs.

### Egg covering

Egg covering by *T. pityocampa* females creates a microhabitat to protect the eggs from adverse climatic conditions (mostly by facilitating heat conservation), as well as from natural enemies, especially parasitoids (Pérez-Contreras and Soler 2004). In the WPs, the egg scales are round shaped and imbricated, forming a regular, tight roof (Fig. 5). By contrast, in the Leiria SP, the scales are smaller, thinner and have a pronounced triangular shape. As the scales curl inwards, a tight roof type adjustment is precluded, leaving some free space among them (Fig. 5) increasing the egg exposure to parasitoids. Although this trait might not be adaptive and could well have resulted from chance alone, following the founder effect that originated the Leiria SP, apparently no counter-selection is acting, as egg parasitism is extremely low in this population (see below). The scales of the SP are also significantly darker than those of the two studied winter populations. A darker color may contribute to raise egg temperature, and to some extent, counteract the disadvantages of a cover made of thinner scales, while temperature is an important factor as the embryos of the SP experience cooler temperatures in May, than those of the WP in August and September. Further research is needed to test this hypothesis of local adaptation.

### Egg parasitism

Three species of egg parasitoids were found in both Leiria populations, SP and WP. Only the parasitoids that emerged in the laboratory and not those previously emerged in the field were recorded. Present results refer to the late emerging parasitoids of the first generation and to the second generation.

In contrast with the WP, a high proportion of generalist species (*T. embryophagum* and *O. pityocampae*) was recorded from the SP, while the specialist parasitoid *B. servadeii* was found at a very low frequency (4.9%). Specialist parasitoids must closely synchronize their life cycles with that of their host (Godfray et al. 1994; Hawkins et al. 1997; Van Nouhuys and Lei 2004), and their patterns of emergence and foraging are strongly constrained by host phenology. On the contrary, emergence and foraging patterns of generalist species can be distributed over longer periods of time. Accordingly, the generalist parasitoids were more efficient than the specialist *B. servadeii* in exploiting the new resource formed by the SP. Our results further indicate that the generalist parasitoids showed partly synchronized emergences with the male flight of Leiria SP (Fig. 3). By contrast and as expected, the activity of the specialist parasitoid *B. servadeii* was well synchronized with the flight period of the winter population, but not with the reproductive period of the SP.

In general, *T. pityocampa* egg parasitoids prefer smaller clutches with larger eggs (Pérez-Contreras and Soler 2004), suggesting that parasitism may also affect the trade-off between the number of eggs and egg size. In fact, over 8 years of sampling, the mean parasitism rate for the Leiria SP remained low, varying from 0 in 2011 to 4.9% in 2004. Nonetheless, a low rate of parasitism was also observed in the Leiria WP, suggesting an overall low abundance of parasitoids in this area. A relationship between parasitism rates and host population density is expected to occur, as observed for other host–parasitoid systems (May et al. 1981), so that future studies, addressing the density dependence issue, between *T. pityocampa* and the parasitoid complex, would be pertinent. Our field surveys based on male trap catches, showed that whereas the Leiria SP has regularly attained relatively high densities during the observed period (between 8.8 and 20.0 males caught per trap), the sympatric Leiria WP registered very low densities (between 0.4 and 1.2 males per trap). Yet, parasitism rates remained low in both the SP and the WP, suggesting that the population densities did not affect parasitism rates over the studied period. On the other hand, the dispersion of the parameter rate of parasitism was highest in the Leiria SP, when compared with all other populations (Fig. 1). This outcome results from a few egg masses only having high parasitism rates. Apparently, although the eggs of the Leiria SP are exposed to a low parasitism pressure, whenever a female parasitoid finds an egg batch, this resource is intensively exploited.

## Conclusions

Rapid morphological differentiation and adaptation to local environments have been documented for species invading new habitats (Lambrinos 2004). Here, we documented the occurrence of rapid differentiation of the phenotypic characteristics and life-history traits for a population experiencing sympatric differentiation due to a shift in the reproductive period, while remaining in situ, using the same habitat and host plant.

Present data demonstrate that the SP of the pine processionary moth, discovered recently in Leiria, exhibits a marked phenotypic divergence in some reproductive traits in relation to the sympatric WP, from which the founders probably originated. As the SP has probably recently evolved (Santos et al. 2007, 2011a), results show that several traits, namely egg size/fecundity/egg covering, now differ between the SP and the sympatric WP, which suggests a rapid differentiation. Moreover, high intrapopulation phenotypic variability was found in the morphological traits of the egg scales (Figs. 4 and 5). If in this case, evolution was due to adaptation, phenotypic plasticity, drift, or a combination of these processes could not be clarified.

Our data further agree with the hypothesis that divergence might be strengthened by exposure to new ecological pressures, now experienced by the SP (Santos et al. 2011b). As a result, a tendency for a shift in the bionomic strategy of the SP is suggested, resulting from a higher energetic investment allocated to survival in relation to reproduction. This is in agreement with (Soberón 2010), who identified bionomic effects as fundamental to explain species distribution at site and local scale. This work therefore provides an insight of how ecological divergence becomes a driving force in the process of allochronic reproductive isolation that is a rare glimpse of sympatric speciation in action.
